# Supplementary material for: The Role of β-Core Fragment hCG in Embryo Implantation and Early Pregnancy
Source: Int J Mol Sci. 2025 Aug 18;26(16):7974. doi: 10.3390/ijms26167974 (PMC12387010; doi:10.3390/ijms26167974)
Supplement: Supplementary file 1 [file ijms-26-07974-s001.zip › ijms-3766433-supplementary.pdf]

Supplement

Methods

S1 ELISA analysis for Anti-bcF-hCG Antibody specification

To perform the ELISA, 96-well immunoplates were initially coated with 100 μL of various target antigens, including Beta core fragment hCG (NIBSC code 99/708), Intact hCG (NIBSC code 18/244), Beta subunit hCG (NIBSC code 75/551), and Alpha subunit hCG (NIBSC code 99/720), all diluted in PBS. This coating process occurred at 37°C for one hour. Following antigen coating, the plates underwent a blocking step at 37°C for two hours using a 0.1% Casein in PBS solution. Subsequently, the plates were washed three times with 0.05% Tween20(BR1706531; Bio-Rad, Hercules, CA, USA) in PBS. For the antibody reaction, anti-bcf-hCG antibody (clone 2G12) was serially diluted starting from 1 μg/mL and 100 μL was dispensed into each well, followed by a one-hour incubation at 37°C. Another washing step, identical to the previous one, was then performed. The detection antibody reaction involved adding 100 μL/well of Goat anti-mouse IgG-HRP, diluted 1:10,000 in a dilution solution (0.05% BSA, 5% Sucrose in PBS), and incubating at 37°C for one hour. A final washing step with 300 μL/well of 0.05% Tween20(BR1706531; Bio-Rad, Hercules, CA, USA) in PBS was conducted three times. For substrate development, 100 μL/well of TMB substrate was added and allowed to react at room temperature for 10 minutes. The reaction was then stopped by adding 100 μL/well of 0.2N H_2SO_4 solution. Finally, the absorbance was measured at 450 nm using an ELISA reader (Epoch™ Microplate Spectrophotometer; BioTek, Winooski, VT, USA) for plate reading.

Table S1. Primer sequences used in real-time quantitative PCR.

Primer name	Forward primers (5'-3')	Reverse primers (3'-5')
RPL7	GCAGATGTACCGCACTGAGATTC	ACCTTTGGGCTTACTCCATTGATA
Hoxa10	CTGAATGGGCTGGGTTTGCT	CCGGCTAAGTTTACCCAGGG
Hoxa11	GGGGATAGGAGCAGGAGGAA	TCTCCCTTTTCACAGCCACC
DEDD	TCACCTACCTCGACGCATTC	CCCACAGCTTGCTTGAGAGA
Cyclin D3	TTTCCCTCCCTTCTTCCCCT	CCCAACCTAACCCTGCTCTG
Cdk4	ATGGCTGCCACTCGATATGAA	TCCTCCATTAGGAACTCTCACAC
Cdk6	GGCGTACCCACAGAAACCATA	AGGTAAGGGCCATCTGAAAACT
EGR	GAGCGAACAACCCTATGAG	GTCGTTTGGCTGGGATAA
Pigf	CAGCCAACATCACTATGCAG	GGGTGACGGTAATAAATACG
Hand2	CAGATGCAGACCCAGGACTC	GGGCAGAAAGAAGGGGAAGG
Msx1	AAGATGCTCTGGTGAAGGCC	TCTTGTGCTTGCGTAGGGTG

Table S2. ELISA data of anti-bcf Antibody specification.

2G12 (anti-bcf-hCG Mab)	Coating Antigen			
Antibody conc.	Intact hCG	**bcf-hCG**	free beta hCG	alpha hCG
1ug/ml	0.008	**0.212**	0.001	0
0.5ug/ml	0.006	**0.164**	0.001	0.001
0.25ug/ml	0.006	**0.141**	0	0
0.125ug/ml	0.003	**0.104**	0	0.005
